# Tuberculosis before and during COVID-19 Pandemic, United States, 2010–2023

**DOI:** 10.3201/eid3203.251459

**Published:** 2026-03

**Authors:** Pei-Jean I. Feng, Christina R. Phares, Robert Pratt, Julie L. Self

**Affiliations:** Centers for Disease Control and Prevention, Atlanta, Georgia, USA

**Keywords:** tuberculosis and other mycobacteria, COVID-19, SARS-CoV-2, severe acute respiratory syndrome coronavirus 2, respiratory infections, viruses, epidemiology, public health surveillance, United States

## Abstract

After a steady decline in tuberculosis (TB) during 2010–2019, the United States reported a sharp drop in 2020 and increases during 2021–2023. We assessed whether TB cases during 2020–2023 differed from what was expected in the absence of the COVID-19 pandemic. Using data from the Centers for Disease Control and Prevention National TB Surveillance System and Electronic Disease Notification system, we constructed Poisson regression models to predict frequencies of TB cases, persons receiving TB diagnosis within 1 year of arrival, and persons for whom postarrival TB follow-up was recommended on the basis of 2010–2019 trends. We observed lower than predicted TB cases (7,170 observed, 8,822 predicted), persons receiving diagnosis within 1 year of arrival (208 observed, 259 predicted), and persons with class B TB (4,827 observed, 7,169 predicted) in 2020. Migration changes and COVID-19–related factors likely contributed to the decrease in TB in 2020 and increases during 2021–2023.

In the United States, tuberculosis (TB) cases and incidence rates (cases/100,000 persons) declined steadily during 1992–2019 (*1*). During 2010–2019, both case counts and incidence rates decreased by an average of 3% per year. In 2020, concurrent with the onset of the COVID-19 pandemic, the United States reported 7,170 TB cases and an incidence rate of 2.2, representing a 19% decline in cases and 20% decline in incidence rate compared with 2019. The percentage decreases in TB case count and incidence rate from 2019 to 2020 were >6–8 times that of the yearly average from 2010–2019 (*1*). The drop in cases in 2020 was likely a result of multiple factors associated with the COVID-19 pandemic, including delayed diagnoses, changes in movement or travel of non–US-born persons to the United States (migration), and pandemic mitigation efforts (*2*).

Since 2001, most TB cases in the United States have occurred among persons born outside the United States (*1*). When stratified by the number of years since arrival, the highest percentage of TB diagnoses among non–US-born persons occurs in the first few years after arrival in the United States. In 2020, a total of 28% of cases among non–US-born persons were diagnosed within 5 years of arrival in the United States, compared with 30% in 2019 and a median of 32% during 2010–2018 (*1*).

TB cases and incidence rates have increased every year since 2020. TB cases increased by 10% from 2020 (n = 7,170) to 2021 (n = 7,866), 6% from 2021 to 2022 (n = 8,332), and 16% from 2022 to 2023 (n = 9,633). Similarly, TB incidence rate increased by 10% from 2020 (2.2 cases/100,000 persons) to 2021 (2.4 cases/100,000 persons), 6% from 2021 to 2022 (2.5 cases/100,000 persons), and 15% from 2022 to 2023 (2.9 cases/100,000 persons) (*1*).

We used multiple data sources to determine if the substantial drop in TB cases in 2020 was statistically different from what would have been expected had the COVID-19 pandemic not occurred and to examine possible factors that contributed to that drop and the subsequent increase in cases after 2020. The Centers for Disease Control and Prevention (CDC) reviewed this activity and deemed it not research. We conducted the study consistent with applicable federal law and CDC policy (*2*).

## Materials and Methods

The 50 US states and the District of Columbia are required to report each new TB case to CDC’s National Tuberculosis Surveillance System (NTSS) (*3*), using the Report of Verified Case of Tuberculosis (*4*). For our analysis, we obtained verified TB case counts from NTSS (*1*,*5*). We created a Poisson regression model to estimate annual TB case counts during 2010–2019, the years preceding the COVID-19 pandemic, which we used to predict counterfactual annual case counts during the pandemic (2020–2023). We used the bootstrap method to obtain robust estimates of the predicted case counts and corresponding prediction intervals by running the Poisson model on 1,000 replicate datasets randomly sampled, with replacement, from the observed 2010–2019 case counts. The median of the 1,000 replicate predictions represents the bootstrap estimate of the predicted annual case count. The 2.5th and 97.5th percentiles represent the 95% prediction interval (PI), which accounts for the uncertainty from estimating the model and the variance of the projected case counts. We applied the bootstrap method to all models in our analysis.

The Poisson model consisted of predictors for case count year: the previous year’s TB case count, which controls for autocorrelation and represents potential lag effects carried over from the previous year, and an offset variable to represent the US population from the US Census Bureau national population totals from 2023 (*6*). The offset variable accounted for population changes and enabled us to model rates, while maintaining the count structure of our data. We also stratified the Poisson model by origin of birth to examine if the association between COVID-19 and TB case counts differed among US-born persons and non–US-born persons. In NTSS, persons born in the United States, in US territories, or elsewhere to >1 US citizen parent were categorized as US-born. All other persons were categorized as non–US-born. We excluded persons with unknown origin of birth from the stratified analysis. We used annual US-born and non–US-born population estimates from the US Census Bureau American Community Survey (ACS) as offsets for the respective stratified Poisson models (*7*). Persons with a value for the citizenship status variable in ACS of not a citizen of the US or US citizen by naturalization were categorized non–US-born; all others were categorized as US-born (*8*).

To investigate the effect of migration-related factors on TB during the COVID-19 pandemic, we predicted the number of persons who received a TB diagnosis within 12 months of arrival to the United States (persons with first-year diagnoses) during 2020–2023 using a Poisson regression, based on 2010–2019 data, limited to non–US-born persons only. We determined the duration between arrival in the United States and diagnosis of TB by calculating the difference between the arrival date and the date the case was first reported to the health department. To control for autocorrelation, the model included predictors representing year of arrival to the United States and the number of persons with first-year diagnoses during the previous year. The offset variable for this model represented the annual number of non–US-born persons, regardless of legal status, who arrived in the United States, as reported by ACS (*7*). Because persons who arrive in the United States may later emigrate from the United States, the number of persons who report arriving during a particular year will decrease over time. We found that the reported number of persons who arrived in the United States in a particular year peaked in the 1-year ACS estimate of the following year. Therefore, we included the ACS estimates from the following year as the offset variable. We used that model to predict the number of persons with first-year diagnoses during 2020–2022. Because the peak number of persons who arrived in 2023 was not published at the time of our analysis, we predicted counts through 2022.

To further assess migration-related factors on TB during the pandemic, we analyzed health information reported to CDC’s Electronic Disease Notification (EDN) system from overseas medical examinations of refugees, immigrants, and some parolees (*9*). Refugees are persons unable or unwilling to return to their country of nationality because of persecution or well-founded fear of persecution (*10*). Immigrants are foreign nationals with an immigrant visa who become lawful permanent residents upon admission to the United States (*12*). Parolees are persons granted parole into the United States for humanitarian reasons or substantial public benefit (*11*). An overseas medical examination, including screening for TB disease, is required for all refugees, all immigrants who apply for their immigrant visa from outside the United States, and some parolees; we collectively refer to those persons as persons screened overseas. Persons assigned a class B TB classification during the overseas evaluation are recommended to have a postarrival evaluation for TB (*13*). Class B TB classifications are assigned to persons who recently finished directly observed treatment for TB disease; have signs or symptoms or chest radiographs suggestive of TB disease, or have known HIV infection with negative sputum cultures and no clinical diagnosis of infectious TB disease; have extrapulmonary TB disease with a normal chest radiograph and negative sputum cultures; have a diagnosis of latent TB infection; or are contacts to infectious TB disease cases (*13*). We examined data from EDN to measure the effects of COVID-19 on the annual number of persons screened overseas who were assigned a class B TB classification (persons with class B TB). We created a Poisson model to predict the number of persons with class B TB during 2020–2023 on the basis of prepandemic data, using year of arrival as the only predictor. We did not include a lag term because the number of persons with class B TB each year was not influenced by the previous year’s number. We used the total number of refugees documented in EDN, along with the number of new arrivals obtaining lawful permanent resident status provided by the US Department of Homeland Security, as the offset variable for this model (*14*).

We conducted statistical analyses using SAS version 9.4 (SAS Institute, Inc, https://www.sas.com) and R statistical software version 4.4.2 (The R Project for Statistical Computing, https://www.r-project.org). We designated statistical significance at 5% (α <0.05). We considered as statistically significant any observed counts during 2020–2023 that fell outside the 95% PIs of the predicted counts.

## Results

During 2010–2023, a total of 129,123 TB cases were reported to NTSS; of those, 96,122 cases were reported in 2010–2019, before the pandemic, and 33,001 cases in 2020–2023, during the pandemic (Table 1). The percentage of TB cases that occurred among US-born persons decreased from 33% (n = 32,083) during 2010–2019 to 26% (n = 8,599) during 2020–2023. The most common age group was 25–44 years among all persons across the prepandemic and pandemic periods: 29,670/96,122 (31%) persons during 2010–2019 and 9,841/33,001 (30%) persons during 2020–2023. The most common age group among US-born persons was 45–64 years, 11,256/32,083 (35%) persons during 2010–2019 and 2,501/8,599 (29%) persons during 2020–2023. 

**Table 1:** Demographic characteristics of all persons with tuberculosis disease reported to the National TB Surveillance System, United States, 2010–2023*

Characteristic	No. (%) persons	
	2010–2019	2020–2023
All	96,122	33,001
Sex		
M	58,544 (61)	20,337 (62)
F	37,567 (39)	12,653 (38)
Age group, y		
0–4	2,627 (3)	755 (2)
5–14	2,003 (2)	706 (2)
15–24	9,599 (10)	3,227 (10)
25–44	29,670 (31)	9,841 (30)
45–64	29,655 (31)	9,608 (29)
>65	22,564 (23)	8,861 (27)
Race or ethnicity†		
American Indian or Alaska Native	1,185 (1)	387 (1)
Asian	31,665 (33)	11,066 (34)
Black or African American	20,814 (22)	5,850 (18)
White	13,129 (14)	3,401 (10)
Native Hawaiian or other Pacific Islander	896 (1)	532 (2)
Hispanic or Latino	27,610 (29)	10,909 (33)
Multiple race	586 (1)	549 (2)

*2010–2019 is the period before the COVID-19 pandemic; 2020–2023 is the period during the COVID-19 pandemic. †Persons who identified as Hispanic or Latino were categorized as Hispanic, regardless of self-reported race. Persons who did not identify as Hispanic or Latino were categorized by self-reported race; if >1 race was reported, the person was categorized as multiple race.

 The most common age group among non–US-born persons was 25–44 years, 22,727/63,990 (36%) persons during 2010–2019 and 7,795/24,341 (32%) during 2020–2023) (Table 2). Among persons with first-year diagnoses, 6,147 (67%) persons arrived in the United States during 2010–2019, before the onset of COVID-19, whereas 3,053 (33%) arrived during and after the pandemic, during 2020–2023 (Table 3). Similar to all non–US-born persons with TB, the most common age group in both periods among persons with first-year diagnoses was 25–44 years (2,425/6,147 [39%] during 2010–2019 and 1,413/3,053 [46%] during 2020–2023). The next most common age group was 15–24 years (1,390/6,147 [23%] during 2010–2019 and 674/3,053 [22%] during 2020–2023) (Table 3).

**Table 2:** Demographic characteristics of persons with tuberculosis disease reported to the National TB Surveillance System, by origin of birth, United States, 2010–2023*

Characteristic	US-born, no. (%) persons			Non–US-born, no. (%) persons	
	2010–2019	2020–2023		2010–2019	2020–2023
All	32,083	8,599		63,990	24,341
Sex					
M	20,668 (64)	5,384 (63)		37,839 (59)	14,914 (61)
F	11,414 (36)	3,213 (37)		26,142 (41)	9,421 (39)
Age group, y					
0–4	2,314 (7)	680 (8)		313 (0)	75 (0)
5–14	1,240 (4)	442 (5)		763 (1)	263 (1)
15–24	2,948 (9)	951 (11)		6,650 (10)	2,275 (9)
25–44	6,932 (22)	2,037 (24)		22,727 (36)	7,795 (32)
45–64	11,256 (35)	2,501 (29)		18,378 (29)	7,090 (29)
>65	7,392 (23)	1,986 (23)		15,156 (24)	6,842 (28)
Race or ethnicity†					
American Indian or Alaska Native	1,176 (4)	382 (4)		9 (0)	5 (0)
Asian	1,331 (4)	442 (5)		30,325 (47)	10,614 (44)
Black or African American	12,148 (38)	2,892 (34)		8,664 (14)	2,953 (12)
White	10,254 (32)	2,351 (27)		2,871 (4)	1,043 (4)
Native Hawaiian or Other Pacific Islander	299 (1)	180 (2)		596 (1)	352 (1)
Hispanic or Latino	6,595 (21)	2,183 (25)		20,986 (33)	8,691 (36)
Multiple race	214 (1)	111 (1)		370 (1)	437 (2)

*2010–2019 is the period before the COVID-19 pandemic; 2020–2023 is the period during the COVID-19 pandemic. †Persons who identified as Hispanic or Latino were categorized as Hispanic, regardless of self-reported race. Persons who did not identify as Hispanic or Latino were categorized by self-reported race; if >1 race was reported, the person was categorized as multiple race.

**Table 3:** Demographic characteristics of persons with first-year tuberculosis diagnoses as reported to the National TB Surveillance System, United States, 2010–2023*

Characteristic	No. (%) persons	
	2010–2019	2020–2023
All	6,147	3,053
Sex		
M	3,583 (58)	1,979 (65)
F	2,564 (42)	1,074 (35)
Age group, y		
0–4	174 (3)	41 (1)
5–14	276 (4)	99 (3)
15–24	1,390 (23)	674 (22)
25–44	2,425 (39)	1,413 (46)
45–64	1,073 (17)	482 (16)
>65	809 (13)	344 (11)
Race or ethnicity†		
American Indian or Alaska Native	0	2 (0)
Asian	2,401 (39)	705 (23)
Black or African American	1,578 (26)	564 (18)
White	268 (4)	194 (6)
Native Hawaiian or Other Pacific Islander	55 (1)	28 (1)
Hispanic or Latino	1,751 (28)	1,469 (48)
Multiple race	60 (1)	20 (1)

*First-year diagnosis is a TB diagnosis within 12 months of arrival to the United States. 2010–2019 is the period before the COVID-19 pandemic; 2020–2023 is the period during the COVID-19 pandemic. †Persons who identified as Hispanic or Latino were categorized as Hispanic, regardless of self-reported race. Persons who did not identify as Hispanic or Latino were categorized by self-reported race; if >1 race was reported, the person was categorized as multiple race.

During 2010–2019, the number of persons with first-year diagnoses fluctuated from 522 to 708, representing an annual percentage change that ranged from −20% to 13% (Table 4). The number of persons with first-year diagnoses in 2020 was 60% lower than in 2019 (n = 208), followed by increases of 227% (n = 680) in 2021 and 70% (n = 1,159) in 2022. Prepandemic, the annual percentage change in the number of non–US-born persons arriving in the United States was −17% to 17% (Table 4). In 2020, the number of non–US-born persons who arrived in the United States decreased by 49% (n = 694,831) from 2019 (n = 1,369,247) before increasing by 110% (n = 1,456,907) in 2021 and 40% (n = 2,036,805) in 2022 (Table 4). The total number of persons with first-year diagnoses and the total number of non–US-born persons who arrived in the United States in 2023 were unavailable at the time of our analysis.

**Table 4:** Percentage change in non–US-born persons with first-year diagnoses of tuberculosis reported to the National TB Surveillance System, by year of arrival, United States, 2010–2023*

Year of arrival	No. persons with first-year diagnoses	Total population†	Percentage change	
			Among persons with first-year diagnoses	Among total population
2010	663	1,158,774	Referent	Referent
2011	558	1,083,782	−15.8	−6.5
2012	631	1,212,664	13.1	11.9
2013	634	1,277,674	0.5	5.4
2014	637	1,494,121	0.5	16.9
2015	672	1,617,277	5.5	8.2
2016	708	1,746,695	5.4	8.0
2017	565	1,447,393	−20.2	−17.1
2018	557	1,341,551	−1.4	−7.3
2019	522	1,369,247	−6.3	2.1
2020	208	694,831	−60.2	−49.3
2021	680	1,456,907	226.9	109.7
2022	1,159	2,036,805	70.4	39.8

*Population consisted of persons not born in the United States, in US territories, or elsewhere to >1 US citizen parent who received a TB diagnosis within 12 months of arrival to the United States. Data for 2023 were not available at the time of analysis. NA, not available. †Population data obtained from the American Community Survey (*7*).

Of persons screened overseas, both before and during the pandemic, 4% were assigned class B TB classifications documented in EDN (Table 5). Before the pandemic, the annual number of persons with class B TB was 18,482–28,529; percentage change fluctuated from −19% to 19%. Compared with 2019, the number of persons with class B TB in 2020 decreased by 74% (n = 4,827), then increased by 129% (n = 11,056) in 2021, 80% (n = 19,905) in 2022, and 29% (n = 25,736) in 2023. During 2010–2019, the total number of persons screened overseas each year was 497,255–729,397. In 2020, the total decreased by 62% (n = 191,536) compared with 2019 (n = 497,255), before increasing by 104% (n = 391,138) in 2021, by 38% (n = 541,145) in 2022, and by 18% (n = 640,549) in 2023 (Table 5).

**Table 5:** Percentage change in persons with class B TB reported to the Electronic Disease Notification system, by year of arrival, United States, 2010–2023*

Year of arrival	No. persons with class B TB	Total population†	Percentage change	
			Among persons with class B TB	Among total population
2010	24,763	574,672	Referent	Referent
2011	22,300	538,061	−9.9	−6.4
2012	26,045	570,436	16.8	6.0
2013	22,884	549,767	−12.1	−3.6
2014	22,059	563,993	−3.6	2.6
2015	24,145	630,993	9.5	11.9
2016	28,529	729,397	18.2	15.6
2017	23,820	604,016	−16.5	−17.2
2018	22,786	554,150	−4.3	−8.3
2019	18,482	497,255	−18.9	−10.3
2020	4,827	191,536	−73.9	−61.5
2021	11,056	391,138	129.0	104.2
2022	19,905	541,145	80.0	38.4
2023	25,736	640,549	29.3	18.4

*Population consisted of persons screened overseas before arrival who were recommended to have a postarrival evaluation for TB in the United States. TB, tuberculosis. †Sum of population data obtained from the Centers for Disease Control and Prevention’s Electronic Disease Notification system (*9*) and the US Department of Homeland Security Yearbook of Immigration Statistics (*15*).

**Figure 1:**
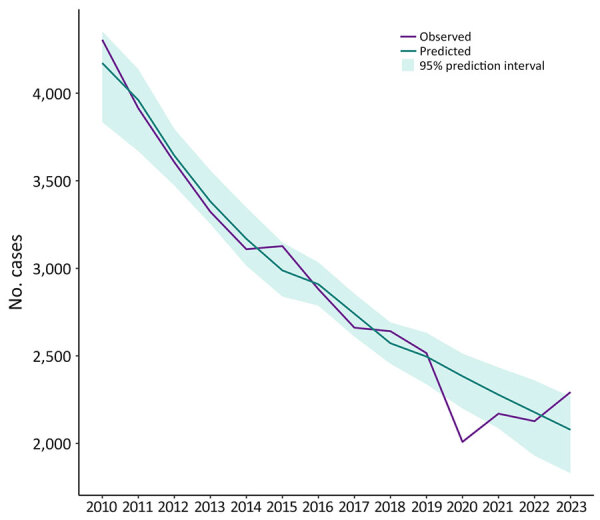
Observed and predicted tuberculosis cases among US-born persons reported to the National TB Surveillance System, United States, 2010–2023. US-born persons are those born in the United States, in US territories, or elsewhere to >1 US citizen parent.

**Figure 2:**
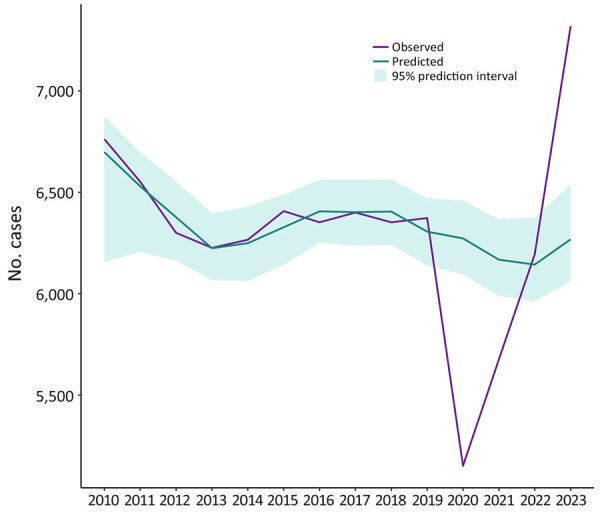
Observed and predicted tuberculosis cases among non–US-born persons reported to the National TB Surveillance System, United States, 2010–2023. Non–US-born persons are those who were not born in the United States, in US territories, or elsewhere to >1 US citizen parent.

On the basis of data reported to NTSS during 2010–2019, the number of TB cases reported in 2020 (n = 7,170) was significantly lower by 19% than the number predicted (8,822 [95% PI 8,560–9,034]) (Table 6; Appendix Figure 1). Among US-born persons, the number of observed cases in 2020 (n = 2,009) was 16% lower than the number predicted (2,384 [95% PI 2,200–2,513]) (Table 6; Figure 1). The number of cases reported among non–US-born persons (n = 5,151) was 18% lower than predicted (6,273 [95% PI 6,096–6,459]) (Table 6; Figure 2). Among all non–US-born persons who arrived in the United States in 2020, a total of 208 persons had first-year diagnoses, which was lower, but not significantly so, by 20% than the predicted count (n = 259 [95% PI 204–294]) (Table 6; Appendix Figure 2). The number of persons with Class B TB who moved to the United States in 2020 (n = 4,827), as documented in EDN, was significantly lower by 34% than what was predicted (7,169 [95% PI 6,705–7,582]) (Table 6; Appendix Figure 3).

**Table 6:** Observed and predicted frequencies of TB cases, persons with first-year diagnoses, and persons with class B TB, United States, 2020–2023*

Category	2020				2023		
	No. observed	No. predicted (95% PI†)	Percentage difference		No. observed	No. predicted (95% PI†)	Percentage difference
All persons‡	7,170	8,822 (8,560–9,034)	−18.7		9,633	8,464 (8,067–8,856)	13.8
US-born persons‡	2,009	2,384 (2,200–2,513)	−15.7		2,293	2,078 (1,830–2,267)	10.3
Non–US-born persons‡	5,151	6,273 (6,096–6,459)	−17.9		7,319	6,268 (6,059–6,535)	16.8
First-year diagnoses§	208	259 (204–294)	−19.7		NA	NA	NA
Persons with class B TB#	4,827	7,169 (6,705–7,582)	−32.7		25,736	22,953 (20,876–25,055)	12.1

*Data for TB cases and first-year diagnoses are from National TB Surveillance System; data for Class B TB are from CDC’s Electronic Disease Notification system (*9*). First-year diagnoses are those of persons who received a diagnosis of TB disease within 12 months of arrival to the United States. Persons with Class B TB are those screened overseas before arrival who are recommended to have a postarrival evaluation for TB in the United States. NA, not available; TB, tuberculosis. †Bootstrap prediction interval. ‡Predicted (counterfactual) frequencies for 2020 and 2023 were estimated from Poisson models using data from NTSS during 2010–2019 (*3*). §Data for 2023 were not available at the time of analysis. #Predicted frequencies for 2020 and 2023 were estimated from a Poisson model using data from EDN during 2010–2019 (*9*).

The observed case count for both US-born and non–US-born persons increased every year during 2020–2023. In 2023, the observed overall case count (n = 9,633) was significantly higher than the predicted count (8,464 [95% PI 8,067–8,856]) by 14% (Tables 6, 7; Appendix Figure 1). The observed numbers of cases in 2023 (US-born, 2,293; non–US-born, 7,319) were also significantly higher than predicted for both the US-born (10%; 2,078 [95% PI 1,830–2,267]) and non–US-born (17%; 6,268 [95% PI 6,059–6,535]) populations (Tables 6, 7; Figures 1, 2). During 2020–2023, the total number of observed cases (n = 33,001) was 5% lower than the sum of predicted cases (n = 34,567) among all persons, 4% lower among US-born persons (observed, 8,599; predicted, 8,918), and 2% lower among non–US-born persons (observed, 24,341; predicted, 24,852) (Table 7).

**Table 7:** Number of observed and predicted tuberculosis cases reported to the National TB Surveillance System, by origin of birth, United States, 2020–2023*

Year	All			US-born			Non–US-born	
	No. observed	No. predicted		No. observed	No. predicted		No. observed	No. predicted
2020	7,170	8,822		2,009	2,384		5,151	6,273
2021	7,866	8,703		2,170	2,278		5,676	6,168
2022	8,332	8,578		2,127	2,178		6,195	6,144
2023	9,633	8,464		2,293	2,078		7,319	6,268
Total	33,001	34,567		8,599	8,918		24,341	24,852

*Predicted (counterfactual) frequencies estimated from Poisson models of NTSS data with predictors representing the year the case was counted, the previous year’s TB case count, and an offset representing the size of the US population from the US Census Bureau. Persons born in the United States, certain US territories, or elsewhere to >1 US citizen parent are categorized as US-born. All other persons are categorized as non–US-born. Persons with unknown origin of birth were excluded from the stratified analysis.

## Discussion

In 2020, with the onset of COVID-19, the TB case count in the United States decreased by 19% compared with 2019 (Table 6). The observed case counts in 2020 among both US-born and non–US-born persons were each significantly lower than predicted by the Poisson model. Several COVID-19-related factors likely contributed to the significant decrease in TB cases in 2020.

Changes in migration likely contributed to the decrease in TB in 2020, because the number of non–US-born persons who entered the United States in 2020 decreased by about half compared with 2019 (*7*). In addition, DHS reported a 62% decrease in the number of new-arrival immigrants in 2020 compared with 2019 (*15*). EDN data also showed a decrease of >60% from 2019 to 2020 in the number of persons arriving in the United States who were screened overseas (Table 5). Fewer persons entered the country in 2020; the number of persons with first-year diagnoses also decreased (*1*). Changes in migration likely contributed to the significant decrease in cases in 2020 but do not fully explain it; we observed a decline in 2020 among US-born persons also.

COVID-19 mitigation strategies might have contributed to the drop in TB cases in 2020. For example, mandated or voluntary sheltering in place, use of masks, and physical distancing might have decreased the transmission of not only COVID-19 but also TB (*16*,*17*). Compliance with shelter-in-place mandates and fear of contracting COVID-19 might also have discouraged people from seeking healthcare (*18*). In a nationwide survey, 41% of adults reported having delayed or avoided medical care because of concerns about COVID-19 (*18*). In addition, by using health insurance claims data, researchers found that overall healthcare use decreased by 23% in March 2020 and by 52% in April 2020 (*19*). In San Francisco, California, USA, the city health department reported a substantial drop in persons seeking medical evaluations for signs and symptoms of TB disease after a legal order for shelter-in-place and a pause on routine medical appointments and elective surgery was issued in March 2020 (*20*).

The diversion of funds and staff away from TB programs and toward the COVID-19 response severely impaired the ability of TB programs to identify and diagnose TB, which likely contributed to a lower-than-expected case count in 2020. Activities such as testing, diagnosing and treating TB, conducting thorough and timely contact investigations, and prompt specimen turnaround time from laboratories were all disrupted (*21*). In April 2020, CDC reported that the deployment of TB program staff to COVID-19 activities had decreased the capacity of TB programs across the country to conduct essential TB-related activities. For example, 52% of TB programs saw a partial or high impact on the ability to diagnose and treat persons with TB disease and 64% saw a partial or high impact on the ability to conduct contact investigations for infectious TB cases (*22*).

Because identifying COVID-19 during the pandemic in emergency settings took priority, and because of the shared characteristics between COVID-19 and TB, such as the mode of transmission, risk factors, and respiratory manifestation, clinicians were likely to overlook the possibility of a TB diagnosis (*21*). Researchers found that during times of elevated COVID-19 incidence, clinicians in California had missed opportunities to diagnose TB disease in persons who sought care for respiratory symptoms by testing only for COVID-19 (*23*).

Since 2021, the number of TB cases in the United States has increased annually. In 2023, the TB case count (n = 9,633) surpassed the count in 2013 (n = 9,238). Similarly, the incidence rate of 2.9 in 2023 had not been reported so low since 2016 (*1*). The percentage increase in the number of persons with first-year diagnoses was >2 times the increase in the total number of non–US-born persons who arrived in the United States from 2020 to and 2021 and 1.8 times higher from 2021 to 2022. Those findings suggest not only that more non–US-born persons arrived in the United States after the onset of the COVID-19 pandemic in 2020 but also that the risk for TB among those persons might have been higher than among persons who arrived before the pandemic, evidenced by the continued increase in the percentage of non–US-born persons with first-year diagnoses (*1*,*7*,*15*). Public health entities could consider expanding prearrival screening to additional groups beyond those currently screened overseas.

COVID-19 mitigation strategies, the diversion of funds and staff from TB programs, and the priority of identifying COVID-19 in 2020 likely caused delayed diagnoses for many TB cases. Therefore, TB cases that occurred in 2020 might not have been identified until 2021 or later, which would have increased the case counts during 2021–2023. Furthermore, delayed diagnoses could have contributed to longer infectious periods, thereby increasing the probability of transmitting TB in the community (*24*).

Although the COVID-19 pandemic seems to have reversed the trend toward TB elimination observed before 2021, our data showed that the cumulative number of observed cases from 2021–2023 was still lower than predicted for that period among both US-born and non–US-born persons. That finding could indicate that the effect of the pandemic on TB in the United States was less pronounced than the year-to-year rise in cases would imply. However, continued attention toward TB elimination is warranted, especially because CDC provisionally reported a fourth consecutive year of increasing TB case counts in 2024 (*25*).

Surveillance data alone did not enable us to distinguish between increased transmission and delayed diagnoses. We could not calculate the exact duration between arrival in the United States and the time of diagnosis because the Report of Verified Case of Tuberculosis does not collect diagnosis date (*4*). Instead, we used the notification date to the health department as a proxy. Our analysis of persons with first-year diagnoses was limited to 2010–2022 because the data for persons who arrived in 2023 were not published at the time of our analysis. Also, we were unable to analyze the demographic characteristics of persons screened overseas because of incomplete EDN data in 2021 and 2022. No official agency counts the number of all persons who arrive in the United States annually, so we approximated the denominator to calculate incidence rate for persons with first-year diagnoses using ACS data, which provides estimates instead of counts.

Our analysis suggests that changes in migration and COVID-19–related factors contributed to both the sharp drop in TB cases in 2020 and the subsequent increase in cases from 2021–2023. In particular, the diversion of resources away from TB prevention and control toward COVID-19 mitigation efforts led to delayed TB diagnoses early in the pandemic, which contributed to the increase in cases after 2020. As time passes, the immediate effects associated with the COVID-19 pandemic will diminish. Changes in migration, however, will continue to influence TB in the United States. To resume progress toward TB elimination, renewed commitment in TB programs is essential to develop TB prevention and control strategies that can withstand challenges brought on by migration changes and future pandemics.

Appendix Additional information about tuberculosis before and during COVID-19 pandemic, United States, 2010–2023.
