# Supplementary material for: Tuberculosis before and during COVID-19 Pandemic, United States, 2010–2023
Source: Emerg Infect Dis. 2026 Mar;32(3):388–96. doi: 10.3201/eid3203.251459 (PMC13016024; doi:10.3201/eid3203.251459)
Supplement: Appendix — Additional information about tuberculosis before and during COVID-19 pandemic, United States, 2010–2023. [file 25-1459-Techapp-s1.pdf]

Article DOI: https://doi.org/10.3201/eid3203.251459

EID cannot ensure accessibility for supplementary materials supplied by authors. Readers who have difficulty accessing supplementary content should contact the authors for assistance.

Tuberculosis before and during COVID-19 Pandemic, United States, 2010–2023

Appendix

Appendix Table. Poisson regression results for models predicting the number of TB cases, persons with first-year diagnoses, and persons with Class B TB, United States*

	Year case counted		Previous year's TB case count	
Category	Coefficient (95% CI)	p value	Coefficient (95% CI)	p value
TB cases among all persons†	-0.01 (-0.02 to -0.003)	0.005	0.0001 (0.00005–0.00009)	<0.001
TB cases among U.S.-born persons‡	-0.03 (-0.05 to -0.01)	0.002	0.0002 (0.00007–0.0002)	<0.001
TB cases among non-U.S.–born persons‡	-0.01 (-0.02 to -0.01)	<0.001	0.0001 (0.00006–0.0001)	<0.001
First-year diagnoses§	-0.05 (-0.05 to -0.04)	<0.001	-0.0067 (-0.001 to -0.0001)	0.01
Persons with Class B TB¶	-0.01 (-0.02 to -0.01)	<0.001	NA#	NA#

*Data for total cases and first-year diagnoses were obtained from the National TB Surveillance System from 2010–2019. Data for Class B TB were obtained from the Electronic Disease Notification system from 2010–2019. NA, not available; TB, tuberculosis.
†Persons diagnosed with TB disease within 12 months of arrival to the United States.
‡Model used data from NTSS during
§Persons diagnosed with TB disease within 12 months of arrival to the United States.
¶Persons screened overseas prior to arrival who are recommended to have a postarrival evaluation for TB in the United States.
#Model did not include previous year's TB case count as a parameter.

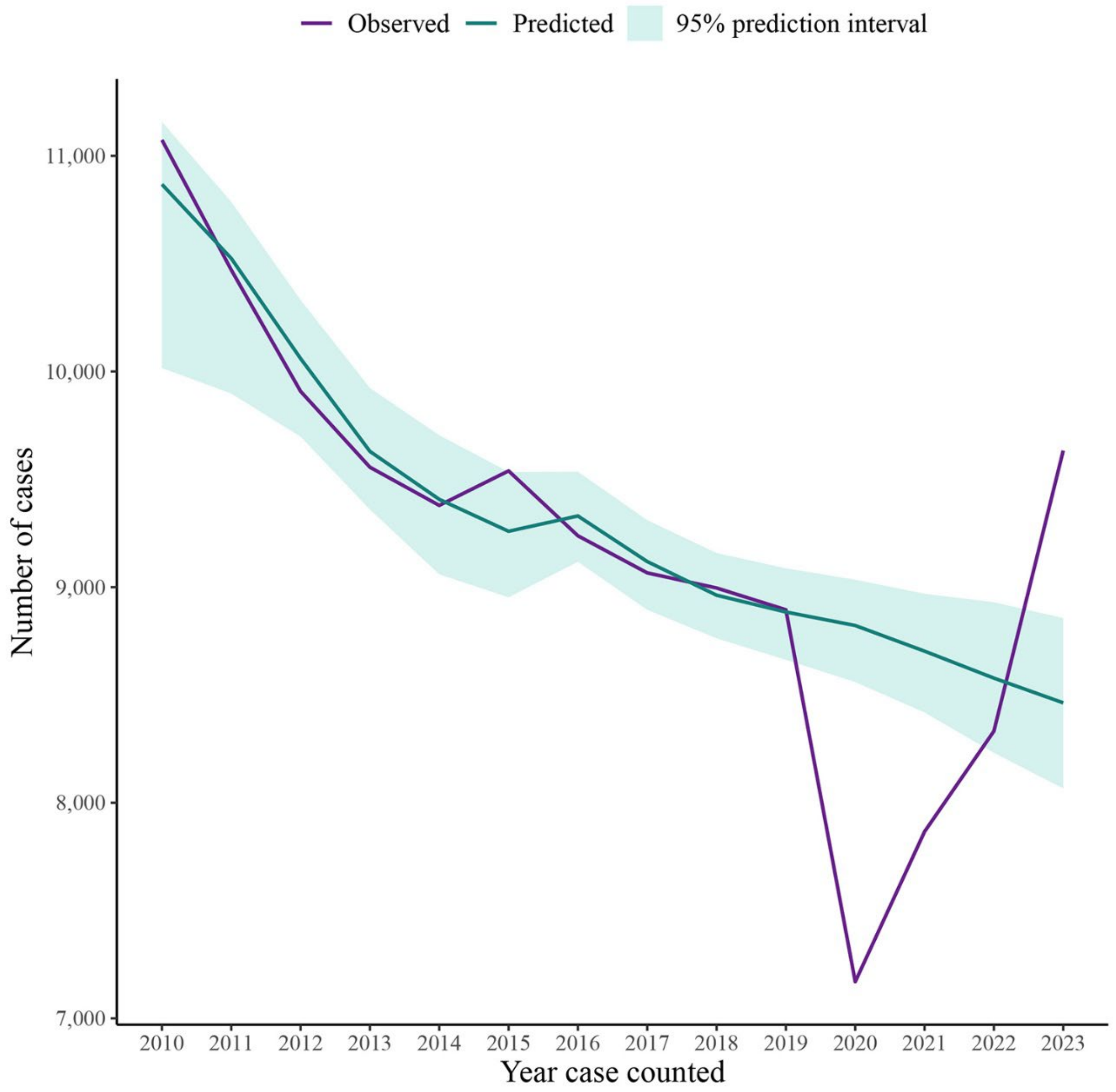

Appendix Figure 1. Number of observed and predicted TB cases among all persons reported to the National TB Surveillance System, 2010–2023.

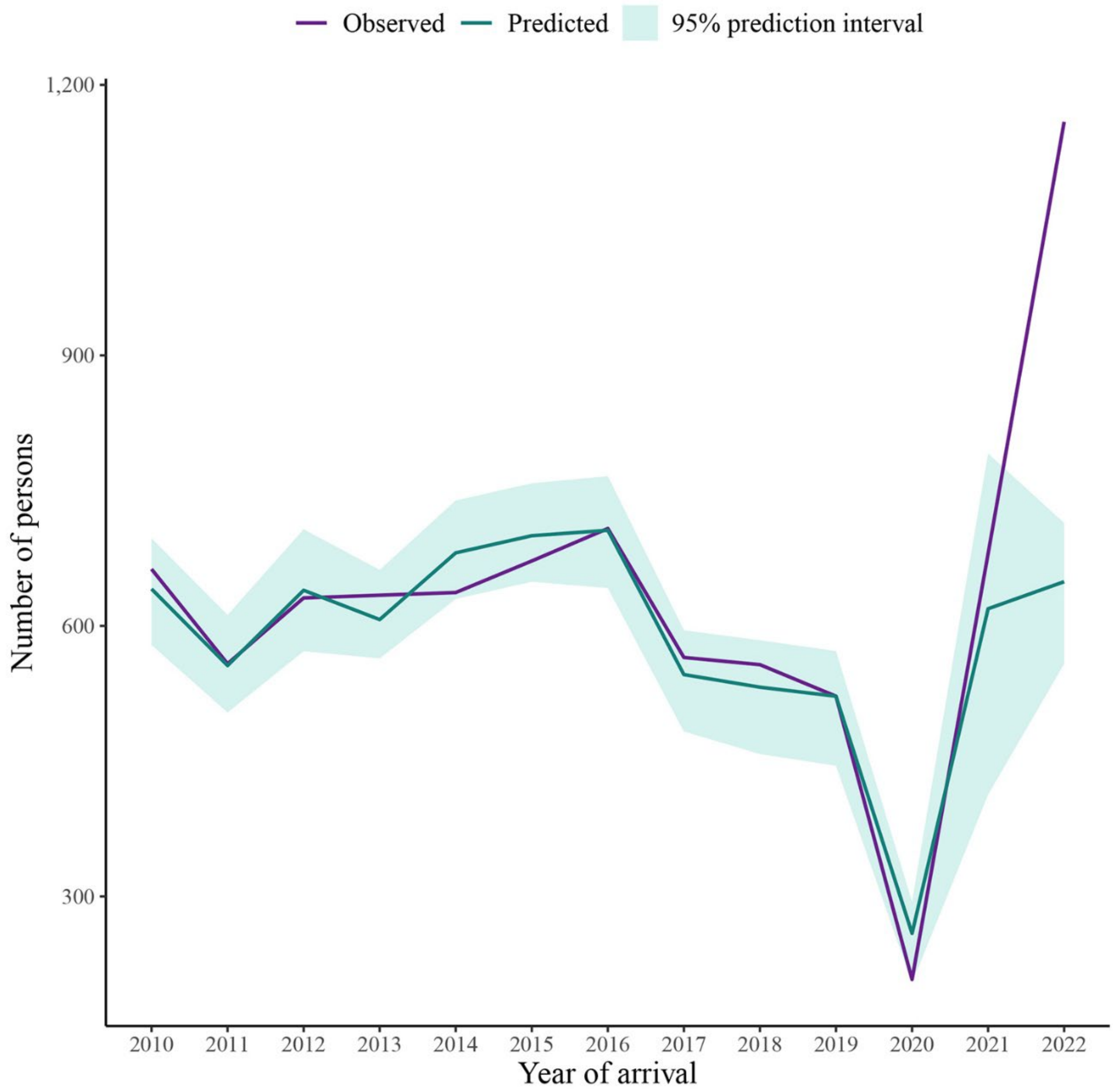

Appendix Figure 2. Number of observed and predicted persons with first-year diagnoses reported to the National TB Surveillance System, 2010–2022. Data for 2023 not shown because the total number of persons with first-year diagnoses who arrived in the United States in 2023 was not available at the time of the analysis.

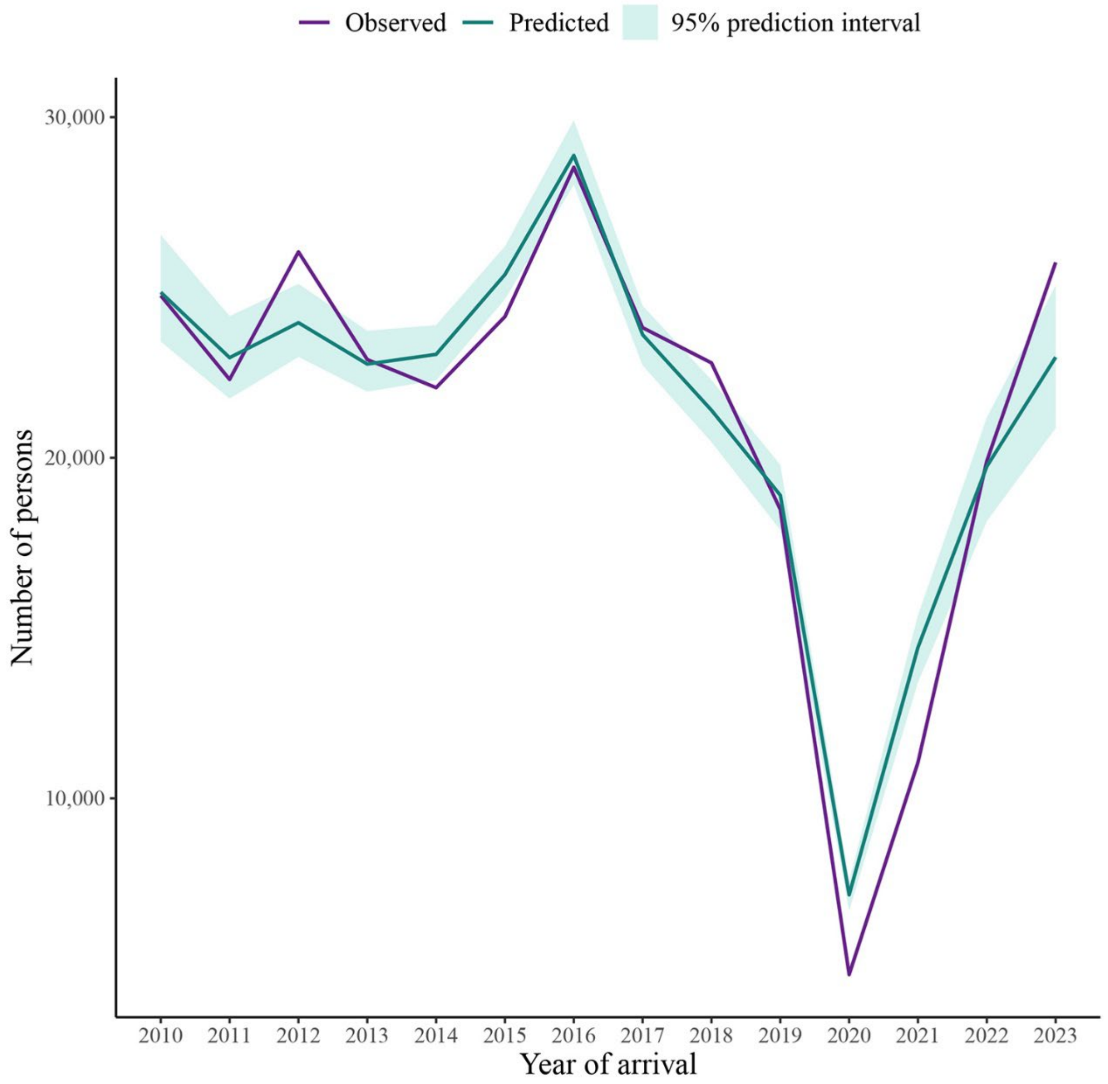

Appendix Figure 3. Number of observed and predicted persons with Class B TB reported to the Centers for Disease Control and Prevention's Electronic Disease Notification system, 2010–2023. Persons screened overseas prior to arrival who are recommended to have a postarrival evaluation for TB in the United States.
